# Investigation entomologique lors de l’épidémie de chikungunya au Tchad en 2020

**DOI:** 10.48327/mtsi.v4i4.2024.619

**Published:** 2024-12-19

**Authors:** Tchonfienet MOUNDAI, Mahamat Alio HAMIT, Israël DEMBA KODINDO, Élise Kalnoné YANGALBE, Hinzoumbé Clément KERAH, Tidjani ABDELSALAM, Sévilor KEKEUNOU

**Affiliations:** 1 Programme national de lutte contre le paludisme, ministère de la Santé publique, Tchad; 2 Laboratoire des recherches, diagnostics et d'expertises scientifiques, Université de N’Djamena, Tchad; 3 Centre hospitalier universitaire La Renaissance, N’Djamena, Tchad; 4 Projet d'appui à la lutte antipaludique au Tchad/Programme des Nations Unies pour le développement, N’Djamena, Tchad; 5 Département de biologie et physiologie animales, Université de Yaoundé 1, Cameroun

**Keywords:** Chikungunya, *Aedes aegypti*, Risque épidémique, Est du Tchad, Abéché, Biltine, Tchad, Afrique subsaharienne, Chikungunya, *Aedes aegypti*, Epidemic risk, Eastern Chad, Abéché, Biltine, Chad, Sub-Saharan Africa

## Abstract

**Observation:**

Le but de lenquête était d'identifier les vecteurs de chikungunya et d’étudier leur bioécologie afin de contribuer aux activités de riposte contre l’épidémie de 2020 dans les villes d’Abéché et de Biltine à l’Est du Tchad.

**Matériel et méthodes:**

Les stades immatures des moustiques *Aedes* ont été collectés et les indices de risque épidémique (indices Récipient, Maison et de Breteau) ont été calculés et comparés par le test du Khi carré. Les larves et nymphes collectées ont été mises en élevage et les adultes qui en sont issus ont été identifiés morphologiquement à l'aide d'une clé dichotomique. La faune résiduelle endophile a été échantillonnée par pulvérisation matinale d'insecticide dans les chambres où seules les femelles étaient collectées.

**Résultats:**

Au total, 2 039 spécimens de moustiques appartenant à trois genres ont été collectés : 470 (23%) ont été identifiés comme *Aedes aegypti,* 731 *Anopheles* spp (36%) et 838 *Culex* spp (41%). Les pots de fleurs étaient les gîtes les plus représentés (69%) suivis des jarres/fûts d'eau (17%) et des pneus/récipients abandonnés (14%). Partout, les indices de risque étaient largement au-dessus des seuils épidémiques définis par l’OMS. Entre les deux villes, seul l'indice Maison a présenté une différence significative (p=0,004) : il était plus élevé à Abéché qu’à Biltine.

**Conclusion:**

L'enquête a permis d'identifier *A. aegypti* comme vecteur probable de chikungunya présent dans les deux villes. Il importe de chercher à comprendre ses comportements de piqûre et de repos ainsi que sa sensibilité aux différentes classes d'insecticides afin d'organiser une lutte antivectorielle efficace.

## Abstract (en)

**Objectives:**

The aim of this study was to identify chikungunya vectors and study their bioecology in order to contribute to the response to the 2020 epidemic in the cities of Abéché and Biltine in eastern Chad.

**Materials and Methods:**

Immature stages of *Aedes* mosquitoes were collected and epidemic risk indices (Container index, House index and Breteau index) were calculated and compared using the Chi-square test. The collected larvae and nymphs were reared, and the resulting adults were morphologically identified using a dichotomous key. Residual endophilic fauna was sampled by morning insecticide spraying in chambers. Only female mosquitoes were collected.

**Results:**

A total of 2,039 mosquito specimens belonging to three genera were collected: 470 (23%) were identified as *Aedes aegypti,* 731 *Anopheles* spp (36%), and 838 Culex spp (41%). Flowerpots were the most common breeding sites (69%), followed by jars/water barrels (17%) *and* abandoned tires or containers (14%). The risk indices were all well above the epidemic thresholds defined by the WHO. Between the two cities, only the house index showed a significant difference (p=0.004): it was higher in Abéché than in Biltine.

**Conclusion:**

The study identified *A. aegypti* as a likely vector of chikungunya in both cities. It is important to understand its biting and resting behavior, as well as its sensitivity to different classes of insecticides, in order to organize effective vector control.

## Introduction

**Figure 1:**
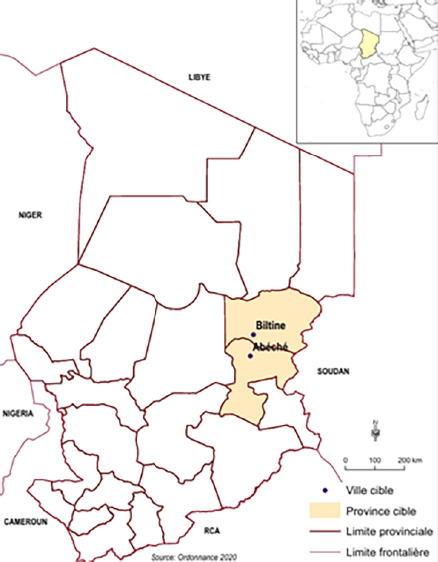
Sites d'enquête (carte Moundai)

Le chikungunya est une arbovirose due à un *Alphavirus* de la famille des Togaviridae [17]. Le virus est transmis à l'homme par des moustiques du genre *Aedes,* majoritairement par les espèces *Aedes aegypti* et *Aedes albopictus,* hautement adaptées aux milieux urbains et péri-urbains [2, 13]. Identifié pour la première fois en Tanzanie en 1952 [20, 21], on en connait actuellement trois génotypes différents : le génotype Ouest africain, le Sud-Est africain et l'asiatique [15]. Le virus du chikungunya (VCHIK) provoque ces dernières années des flambées de plus en plus fréquentes et plus étendues [6]. Depuis 2004, il est devenu un problème majeur de santé publique au monde [9]. Le terme « chikungunya » signifie littéralement « marcher courbé » en langue makondé (parlée en Tanzanie), en référence à l'attitude caractéristique des patients atteints par la maladie [21]. La maladie se manifeste par une fièvre d'apparition brutale accompagnée de douleurs articulaires qui imposent souvent « une marche courbée » aux patients [4]. À l'heure actuelle, on ne dispose pas de traitement antiviral spécifique [1]. Les moyens de lutte les plus vulgarisés incluent la protection individuelle contre les piqûres des moustiques et la destruction de leurs gîtes larvaires les plus évidents [13]. Cependant, les études sur les molécules vaccinales sont prometteuses. Les résultats obtenus avec le vaccin VLA 1553, en essai de phase 3, fait de lui un excellent candidat pour la prévention de l'infection au VCHIK [23]. En plus, un autre candidat, Ixchiq, vient d’être approuvé par la « *Food and Drug Administration »* (FDA) malgré les restrictions dans son utilisation [5]. Avant 2020, le Tchad n'avait jamais déclaré ni importé sur son territoire de cas de chikungunya. En avril 2020, le district sanitaire d’Abéché a commencé à notifier des malades présentant des signes et symptômes rappelant ceux du paludisme, notamment dans la ville d’Abéché (Fig. 1). Les analyses des échantillons de sang total des patients au laboratoire mobile du G5 Sahel utilisant la technique de transcription inverse suivie d'une amplification en chaîne par polymérase (RT-PCR) ont mis en évidence la présence du pathogène VCHIK. Ces résultats ont été confirmés par un contrôle qualité aux laboratoires du Centre Pasteur de Yaoundé (Cameroun), utilisant la même technique. Cela a conduit les autorités politiques à déclarer la toute première épidémie de chikungunya au Tchad le 27 août 2020. Par la suite, d'autres échantillons sanguins prélevés à Biltine (Fig. 1), Goz-Beïda et Abdi, tous à l’Est du Tchad, se sont également révélés positifs. Selon les données non publiées de la surveillance épidémiologique, l’épidémie avait fait près de 40 000 cas au total dont un décès, soit un taux apparent de mortalité de moins de 0,1 %. La plupart des cas ont été enregistrés à Abéché (plus de 30 000 cas) et à Biltine (plus de 6 000 cas).

Aussi, jusqu’à la déclaration de cette épidémie, il n'existait aucune donnée sur les vecteurs de cette arbovirose au Tchad en général, et dans les localités touchées en particulier. Le Programme national de lutte contre le paludisme (PNLP) y avait conduit des évaluations entomologiques focalisées uniquement sur les anophèles, vecteurs du paludisme. Il était donc important de mener des investigations pour comprendre les facteurs déterminants entomologiques de l’épidémie afin de prévoir son évolution et contrôler la propagation du VCHIK dans le pays.

Le présent travail rend compte des activités entomologiques conduites à Abéché et Biltine respectivement en août et septembre 2020, lors de cette épidémie. Le but était de contribuer à la documentation scientifique de l’épidémie de chikungunya à l'est du Tchad. Plus spécifiquement, il était question de rechercher et d'identifier les moustiques vecteurs de chikungunya dans les localités touchées, d’évaluer les indicateurs entomologiques de risque de propagation de la maladie afin de proposer des méthodes de lutte antivectorielle appropriées.

## Méthodologie

### Les localités investiguées

Les investigations ont eu lieu dans les villes d’Abéché (13° 50’ 24’’ Nord, 20° 49’ 48’’ Est) et de Biltine (14° 31’ 39’’ Nord, 20° 55’ 36’’ Est), chefslieux respectifs des provinces du Ouaddaï et du Wadi Fira frontalières du Soudan (Fig. 1). Sur la base du dernier recensement de la population et de l'habitat au Tchad en 2009, la population d’Abéché est estimée à plus de 150 000 habitants contre plus de 25 000 pour Biltine. Les deux villes sont dans le domaine sahélien caractérisé par une courte saison de pluies (juillet à septembre). La moyenne annuelle des précipitations se situe autour de 500 mm, et la température moyenne est de 37°C pour une humidité relative moyenne d'environ 25 % [8].

L'enquête s'est déroulée en août 2020, en pleine saison de pluies à Abéché et juste après les dernières pluies, en septembre, à Biltine. A Abéché, elle a couvert 4 zones de santé (Salamat, Ahmat El Badawi, Kamina et Djatinié) choisies au hasard sur les 11 que compte la ville tandis qu’à Biltine les deux zones (Biltine Est et Biltine Ouest) ont été prospectées.

### Prospection des stades immatures et évaluation du risque épidémique

Les formes immatures (larves et nymphes) *d’Aedes,* ont été recherchées dans les gîtes artificiels rencontrés dans les ménages. L'eau des gîtes positifs a été passée à travers un tamis à maille très fine pour retenir les larves et les nymphes. Ces dernières ont été récupérées et mises en élevage dans des bassines plastiques jusqu’à l'obtention des stades imaginaux. Les larves ont été nourries avec du biscuit commercial et les adultes ont reçu une solution sucrée à 10 % préparée avec du sucre commercial.

Le risque entomologique a été exprimé sur le modèle de la fièvre jaune, par les indices stégomyiens définis par l’Organisation mondiale de la santé (OMS) [16]. Il s'agit de l'indice Récipient (nombre de gîtes positifs par rapport au nombre de gîtes en eau), de l'indice de Breteau (nombre de gîtes renfermant des larves ou nymphes *d’Aedes* spp. pour 100 maisons) et de l'indice Maison (pourcentage de maisons possédant des gîtes positifs). Un gîte est considéré positif quand il renferme au moins une larve et/ou nymphe *d’Aedes* spp. Pendant la prospection des stades immatures, il a été fait usage de la définition de l’OMS selon laquelle une maison est « toute structure dans laquelle dort habituellement au moins une personne » [16].

### Échantillonnage des moustiques adultes

Pour échantillonner les moustiques adultes, nous avons récolté la faune résiduelle endophile par *pyrethrum spray catches* (PSC) qui consiste en une pulvérisation matinale d'insecticide dans les chambres [25]. Pour cela, un insecticide commercial (Oro^®^) de la famille des pyréthrinoïdes et contenant un synergiste (piperonyl butoxide) a été utilisé. Dans la chambre à pulvériser, des draps blancs sont étalés de façon à couvrir entièrement le plancher pour permettre de récolter les moustiques qui tombent sous l'effet de l'insecticide. Seuls les moustiques femelles ont été collectés.

### Identification des spécimens

Les moustiques issus des différentes collectes ont été groupés par genre et seules les espèces du genre *Aedes* ont été identifiées morphologiquement grâce aux clés dichotomiques [11]. Ils ont été regroupés en lots mono spécifiques tout en tenant compte des méthodes, dates et lieux de capture, et aussi de leur état de réplétion.

### Paramètres mesurés

Les variables mesurées pendant l'enquête ont été :

- Indice récipient (IR) = nombre de gîtes positifs * 100 / nombre de gîtes en eau;
- Indice maison (IM) = nombre de maisons avec gîtes positifs * 100 / nombre de maisons visitées;
- Indice de Breteau (IB) = nombre de gîtes positifs *100 / nombre de maisons visitées;
- Nombre de spécimens de *Aedes* femelles collectés dans chaque site.

Selon l’OMS, le risque épidémique est associé à des indices stégomyiens de 4-35 ou plus pour l’IM, 3-20 pour l’IR et 5-50 ou plus pour l’IB [16]. Les indices de risques ont été comparés entre les deux villes en utilisant le test de Khi-carré (seuil de signification de 0,05).

Nous avons obtenu une autorisation administrative du ministère de la Santé publique à travers un ordre de mission. Sur le terrain, la procédure d'enquête a été expliquée aux autorités locales et aux chefs de ménages. La prospection a eu lieu chez les seules personnes consentantes.

## Résultats

Cette enquête entomologique a permis de collecter 2 039 spécimens de moustiques femelles (1 859 par PSC et 180 par élevage des stades immatures), tous genres confondus, dans les deux sites.

### Prospection des stades immatures

Le Tableau I présente les villes prospectées, les gîtes trouvés et les indices de risque épidémique calculés. Au total, 94 ménages, correspondant à 269 maisons, ont été visités et 126 récipients d'eau détectés dont 49 positifs.

**Tableau I:** Prospection des stades immatures et indices de risque épidémique de chikungunya dans les deux villes prospectées

Villes	NMV	NGT	NGP	MGP	Indices stégomyiens		
					IM (%)	IR (%)	IB (%)
Abéché	120	80	35	24	20	44	29
Biltine	149	46	14	12	8	30	9
Total	269	126	49	36	13	39	18

NMV/NHV : Nombre de maisons visitées/Number of houses visited; NGT/NBF : Nombre de gîtes en eau trouvés/Number of breeding sites found; NGP/NPBF : Nombre de gîtes positifs/Number of positive breeding sites; MGP/HPB : Maison avec gîtes positifs/House with positive breeding sites; IM/HI : Indice maison/House Index; IR/CI : Indice récipient/Container Index; IB/BI : Indice de Breteau/Breteau index.

Les larves et nymphes collectées pendant cette prospection ont été mises en élevage et ont permis d'obtenir 180 adultes (111 à Abéché et 69 à Biltine) tous identifiés comme *A. aegypti.*

Les trois indices de risque ont été au-dessus des seuils définis par l’OMS. Ils ont été plus élevés à Abéché qu’à Biltine. Mais seul l'indice maison a présenté une différence significative (p=0,004) entre les deux sites. Il n'y avait pas de différence significative pour les indices récipient (p=0,6) et de Breteau (p=0,13). Les indices stégomyiens dépassent les seuils de l’OMS à Abéché, alors qu’à Biltine, l'indice de Breteau reste inférieur au seuil de l’OMS.

### Typologie des gîtes

**Figure 2:**
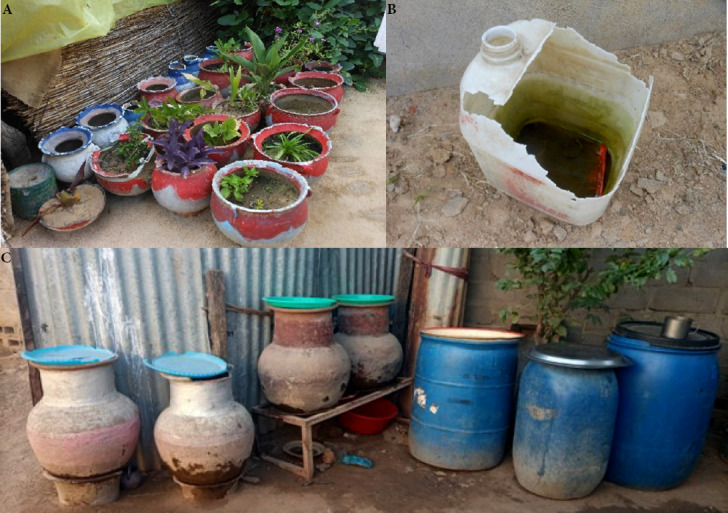
A. Pots de fleurs dans un ménage; B. Bidon abandonné trouvé avec plein de larves d’Aedes *aegypti*; C. Récipients de conservation d'eau

Divers types de gîtes larvaires ont été retrouvés et ceux-ci ont été regroupés en trois catégories : pots de fleurs, récipients de conservation d'eau (jarres et fûts) et objets abandonnés (pneus et récipients) (Fig. 2). Au total, 126 récipients d'eau ont été recensés pendant l'enquête. Les pots de fleurs étaient les plus représentés (69 %) suivis des récipients de conservation d'eau (17 %) et des objets abandonnés (14 %).

### Collecte des moustiques adultes

Au total, 40 chambres ont été pulvérisées à Abéché et 60 à Biltine. Ainsi, 1 859 moustiques femelles, appartenant à trois genres *(Aedes, Anopheles* et *Culex)* ont été collectés par PSC. Les résultats des collectes sont consignés dans le Tableau II. Tous les 290 spécimens *d’Aedes* collectés par PSC ont été identifiés morphologiquement comme l'espèce *A. aegypti.* Ainsi, pour l'ensemble de la collecte (PSC et collecte larvaire), il y avait 470 *A. aegypti,* soit 23 % des femelles collectées.

**Tableau II:** Répartition des moustiques collectés par PSC sur les deux sites

Villes	Nb. chambres pulvérisées	Moustiques femelles collectés			
		*Aedes* spp n (%)	*Anopheles* spp n (%)	*Culex* spp n (%)	Total n (%)
Abéché	40	190 (25)	42 (6)	526 (69)	758 (100)
Biltine	60	100 (9)	689 (63)	312 (28)	1 101 (100)
**Total**	**100**	**290 (16)**	**731 (39)**	**838 (45)**	**1 859 (100)**

n = Nombre de moustiques par genre collectés par ville/Number of mosquitoes by genus collected by city

## Discussion

L'objectif de l'enquête était d'identifier les espèces *d’Aedes* susceptibles de transmettre le VCHIK dans les villes d’Abéché et de Biltine où l’épidémie était déclarée. Les rapports d'activités de la section lutte antivectorielle du PNLP avaient signalé en 2017 et en 2018 la présence des moustiques du genre *Aedes* à Abéché et à Biltine, mais n'avaient donné aucune précision sur les espèces [18, 19]. Notre enquête confirme la présence en nombre remarquable dA. *aegypti* dans les deux localités (23 % des moustiques collectés) où le VCHIK avait déjà été isolé chez des patients sans antécédent de voyage récent. *Aedes aegypti* est l'un des vecteurs majeurs du VCHIK aux côtés d’A. *albopictus* [12]. Sa présence ainsi que l'infection massive des populations font conclure à une transmission autochtone du VCHIK dans ces villes de l’Est du Tchad. Cependant, son rôle vecteur dans les deux villes n'a pas pu être établi faute de matériel de conservation adéquat d’échantillons.

Les pots de fleurs ont représenté plus des deuxtiers des gîtes favorables à la reproduction des *Aedes,* suivis des récipients de conservation d'eau à usage domestique. Sachant que les fleurs sont entretenues tout au long de l'année et que les difficultés d'accès à l'eau obligent les habitants à faire des stocks, on peut penser qu’A. *aegypti* trouvera toujours des gîtes de reproduction même en pleine saison sèche, comme Diarrassouba et Dossou-Yovo [10] l'ont constaté en Afrique de l’Ouest. À Abéché comme à Biltine, la culture des fleurs semble être de règle chez les habitants ne sachant probablement pas que les pots de fleurs constituent des gîtes potentiels de reproduction d’A. *aegypti.* Les ménages ne disposant pas de pots de fleurs sont des exceptions. Une communication pour le changement de comportement de la population serait d'une grande importance pour ralentir la prolifération du vecteur dans la zone. Les tests de sensibilité aux insecticides qui visaient à optimiser la lutte chimique contre les vecteurs n'ont pu être réalisés. Les échantillons des larves d’A. *aegypti* collectés n’étaient pas suffisants (seulement 180 *A. aegypti* femelles issus de la collecte larvaire sur les deux sites) pour les envisager. Dans les deux localités prospectées, les trois indices des risques mesurés ont été largement au-dessus des seuils définis par l’OMS [16]. Mais comme il n'y a pas de surveillance des vecteurs d'arboviroses au Tchad pour lancer l'alerte, cette épidémie a surpris tous les acteurs du système de santé, y compris ceux du service de surveillance épidémiologique. Les indices de risque étaient plus élevés à Abéché qu’à Biltine. En septembre, les pluies avaient cessé de tomber sur la ville de Biltine. Kahamba *et al.* [14] ont montré en Tanzanie que les indices maison et de Breteau diminuaient en saison sèche. On peut penser que l'assèchement de certains gîtes et les facteurs climatiques tels que la température élevée et la faible humidité relative aient joué en défaveur du développement des moustiques à Biltine. En revanche, à Abéché, les investigations ont eu lieu en pleine saison des pluies avec des températures plus basses et une humidité relative plus élevée.

Une faible proportion d’*A. aegypti* adulte a été collectée sur les deux sites (16 %). Deux tiers des femelles *A. aegypti* ont été collectés à Abéché où les chambres ayant servi à la capture ne représentaient que 40 % des chambres prospectées. L'espèce présente généralement une forte tendance à l'endophilie, notamment dans la région néotropicale [22, 24]. Cependant elle peut avoir une tendance plutôt exophile dans certaines zones comme l'ont montré Badolo et *al.* [3] au Burkina Faso. Si c'est le cas dans nos deux sites, la collecte de la faune résiduelle endophile ne pouvait y permettre une bonne évaluation de la densité du vecteur. Ainsi, d'autres méthodes d’échantillonnage comme l'utilisation d'un aspirateur de type *CDC backpack* [7], la capture sur appât humain ou l'utilisation des pièges à appât animal ou attractifs chimiques auraient pu être utilisées. Elles auraient été d'un apport important dans la compréhension du comportement de repos d’A. *aegypti* à l'est du Tchad. Cependant, dans le contexte d'une épidémie confirmée, la capture sur homme aurait été trop risquée et non éthique. Par ailleurs, les pièges ne faisaient pas partie du matériel de collecte en notre possession pendant l'enquête.

Sur notre recommandation, la municipalité d’Abéché a désinsectisé les véhicules en partance vers d'autres villes et une campagne de sensibilisation sur l’élimination des gîtes larvaires potentiels et le respect des mesures de prévention individuelle a été conduite dans les localités touchées.

## Conclusion et perspectives

Lors de cette investigation, nous avons identifié *A. aegypti* comme vecteur probable de la transmission du VCHIK dans les localités visitées. Pour une meilleure lutte antivectorielle, les connaissances sur le comportement des vecteurs et sur leur sensibilité aux insecticides sont primordiales. En raison du nombre insuffisant d’échantillons (seulement 180 *A. aegypti* femelles issues de la collecte larvaire sur les deux sites), les tests de sensibilité n'ont pas pu être réalisés. Aussi, le taux d'infectivité au VCHIK chez les vecteurs n'a pas pu être déterminé faute de matériel de conservation adéquat. D'autres interrogations subsistent, notamment sur la lignée génotypique et la provenance du virus à l'origine de cette épidémie.

## Financement

Ce travail a été réalisé avec l'appui financier du ministère de la Santé publique du Tchad.

## Remerciements

Nous exprimons notre gratitude au ministère de la Santé publique qui, à travers la direction de surveillance épidémiologique, a financé les activités de terrain. Nous remercions les autorités sanitaires provinciales pour leurs appuis administratifs et les relais communautaires pour avoir facilité l'accès dans les ménages.

## Contribution des auteurs et de l'autrice

MOUNDAI Tchonfienet : Conception de l’étude, prospection bibliographique, définition et rédaction de la méthodologie, enquêtes de terrain, analyse des données et interprétation des résultats et rédaction du manuscrit

HAMIT Mahamat Alio : Définition de la méthodologie, analyse des données et corédaction du manuscrit

DEMBA KODINDO Israël : Enquêtes de terrain et corédaction du manuscrit

YANGALBE Kalnoné Élise : Définition de la méthodologie et enquêtes de terrain

KERAH Hinzoumbé Clément : Validation du protocole, analyse des données et relecture et validation du manuscrit

ABDELSALAM Tidjani : Relecture et validation du manuscrit

KEKEUNOU Sévilor : Relecture et validation du manuscrit

## Lien d'intérêt

Les auteurs déclarent n'avoir aucun lien d'intérêt en relation avec le contenu de cet article.
