# Supplementary material for: Entomological investigation during the chikungunya epidemic in Chad in 2020
Source: Med Trop Sante Int. 2024 Dec 19;4(4):mtsi.v4i4.2024.619. [Article in French] doi: 10.48327/mtsi.v4i4.2024.619 (PMC11892385; doi:10.48327/mtsi.v4i4.2024.619)
Supplement: Related texts

# Research article: Entomological investigation during the chikungunya epidemic in Chad in 2020

## Introduction

Chikungunya is an arbovirosis caused by an *Alphavirus* of the family Togaviridae [17]. The virus is transmitted to humans by mosquitoes of the genus *Aedes,* mainly the species *Aedes aegypti* and *Aedes albopictus,* which are highly adapted to urban and peri-urban environments [2, 13]. First identified in Tanzania in 1952 [20, 21], three distinct genotypes are currently known: West African, Southeast African, and Asian [15]. In recent years, chikungunya virus (CHIKV) has caused increasingly frequent and widespread outbreaks [6]. Since 2004, it has become a major public health problem worldwide [9].

The term “chikungunya” literally means “to walk hunched over” in the Makonde language (spoken in Tanzania), referring to the characteristic posture of patients affected by the disease [21]. The disease manifests as a sudden onset of fever accompanied by joint pain, often forcing patients to “walk hunched over” [4]. No specific antiviral treatment is currently available [1]. The most popular control measures include personal protection against mosquito bites and destruction of the most obvious breeding sites [13]. However, studies of vaccine molecules are promising. The results obtained with the VLA1553 vaccine, currently in phase 3 trials, make it an excellent candidate for the prevention of VCHIK infection [23]. In addition, another candidate, Ixchiq, has just been approved by the Food and Drug Administration (FDA) despite restrictions on its use [5].

Prior to 2020, Chad had never reported or imported a case of chikungunya on its territory. In April 2020, the Abéché health district began reporting patients with malaria-like signs and symptoms, particularly in the city of Abéché (Fig. 1). Analysis of the patients’ whole blood samples at the G5 Sahel mobile laboratory using the reverse transcription followed by polymerase chain reaction (RT-PCR) technique revealed the presence of the CHIKV pathogen. These results were confirmed by quality control at the Centre Pasteur laboratories in Yaoundé, Cameroon, using the same technique. This led the political authorities to declare the first Chikungunya epidemic in Chad on August 27, 2020. Subsequently, other blood samples collected in Biltine (Fig. 1), Goz-Beïda and Abdi, all in eastern Chad, were also tested positive. According to unpublished epidemiologic surveillance data, the epidemic involved a total of nearly 40,000 cases, including one death, for an apparent mortality rate of less than 0.1%. Most cases were reported in Abéché (over 30,000 cases) and Biltine (over 6,000 cases).

**Figure 1:** Survey site locations (carte Moundai)

Until the declaration of this epidemic, there was no data on the vectors of this arbovirosis in Chad in general and in the affected localities in particular. The National Malaria Control Program (NMCP) had carried out there entomological studies focusing exclusively on *Anopheles,* the vectors of malaria. Therefore, it was important to carry out studies to understand the entomological determinants of the epidemic in order to predict its evolution and control the spread of CHIKV in the country.

The present work reports on entomological activities carried out in Abéché and Biltine in August and September 2020, respectively, during this epidemic. The aim was to contribute to the scientific documentation of the chikungunya epidemic in eastern Chad. More specifically, the objective was to study and identify the mosquito vectors of chikungunya in the affected localities and to evaluate entomological indicators of the risk of disease spread in order to propose appropriate vector control methods.

## Methodology

### Localities surveyed

The survey took place in the towns of Abéché (13° 50’ 24’’ North, 20° 49’ 48’’ East) and Biltine (14° 31’ 39’’ North, 20° 55’ 36’’ East), the capital cities of Ouaddaï and Wadi Fira provinces, respectively, which border on Sudan (Fig. 1). Based on the most recent population and housing census in Chad in 2009, the population of Abéché is estimated to be over 150,000, compared to over 25,000 for Biltine. Both cities are located in the Sahel zone with a short rainy season (July to September). The average annual rainfall is about 500 mm, and the average temperature is 37°C with an average relative humidity of about 25% [8].

The survey took place in August 2020, at the height of the rainy season in Abéché, and just after the last rain in September in Biltine. In Abéché, the survey covered 4 health areas (Salamat, Ahmat El Badawi, Kamina and Djatinié) randomly selected among the 11 areas of the city, while in Biltine, both areas (Biltine East and Biltine West) were surveyed.

### Survey of immature stages and epidemic risk assessment

Immature forms (larvae and nymphs) of *Aedes* were searched for in artificial breeding sites found in households. Water from positive sites was passed through a fine-mesh sieve to retain larvae and nymphs. Nymphs were collected and reared in plastic basins until they reached the imaginal stage. Larvae were fed commercial biscuits and adults were fed a 10% sugar solution prepared with commercial sugar.

The entomological risk was expressed on the basis of the yellow fever model, using the Stegomyia indices defined by the World Health Organization (WHO) [16]. These are the Container index (number of positive sites in relation to the number of sites in the water), the Breteau index (number of sites containing larvae or nymphs of *Aedes* spp. per 100 houses), and the House index (percentage of houses with positive sites). A house is considered positive if it contains at least one larva and/ or nymph of *Aedes* spp. The WHO definition of a “house” as “any structure in which at least one person customarily sleeps” [16] was used for immature stage sampling.

### Sampling of adult mosquitoes

To sample adult mosquitoes, we collected the residual endophilic fauna by pyrethrum spray catching (PSC), which consists of a morning spray of insecticide in bedrooms [25]. A commercial insecticide (Oro^®^) belonging to the pyrethroid family and containing a synergist (piperonyl butoxide) was used. White sheets were spread out to cover the entire floor so that any mosquitoes that fell under the effect of the insecticide could be collected. Only female mosquitoes were collected.

### Identification of specimens

Mosquitoes from the different collections were grouped by genus. Only species of the genus *Aedes* were identified morphologically using dichotomous keys [11]. They were grouped into monospecific batches, taking into account the methods, dates and places of capture, as well as their state of replenishment.

### Measured parameters

The variables measured during the survey were:

- Container index (CI) = percentage of water-holding containers infested with larvae or pupae;
- House index (HI) = percentage of houses infested with larvae and/or pupae;
- Breteau index (BI) = percentage of positive containers in inspected houses;
- Number of female *Aedes* mosquitoes collected at each site.

According to the WHO, epidemic risk is associated with Stegomyia indices of 4-35 or more for MI, 3-20 for RI and 5-50 or more for IB [16]. The risk indices were compared between the two cities using the chi-squared test (significance level 0.05).

We obtained administrative approval from the Ministry of Public Health through a mission order. In the field, the survey procedure was explained to local authorities and household heads. Only consenting individuals were interviewed.

## Results

The entomological survey collected 2,039 specimens of female mosquitoes (1,859 by PSC and 180 by rearing of immature stages), all genera combined, from the two sites.

### Survey of immature stages

Table I shows the towns surveyed, the breeding sites found, and the epidemic risk indices calculated. A total of 94 households, corresponding to 269 houses, were visited and 126 water containers were detected, of which 49 were positive.

**Table I:** Survey of immature stages and chikungunya epidemic risk indicators in the two cities surveyed

Cities	NHV	NBF	NPBF	HPB	Stegomyia indices		
					HI (%)	CI (%)	BI (%)
Abéché	120	80	35	24	20	44	29
Biltine	149	46	14	12	8	30	9
**Total**	**269**	**126**	**49**	**36**	**13**	**39**	**18**

NMV/NHV : Nombre de maisons visitées/Number of houses visited; NGT/NBF : Nombre de gîtes en eau trouvés/Number of breeding sites found; NGP/NPBF : Nombre de gîtes positifs/Number of positive breeding sites; MGP/HPB : Maison avec gîtes positifs/House with positive breeding sites; IM/HI : Indice maison/House Index; IR/CI : Indice récipient/Container Index; IB/BI : Indice de Breteau/Breteau index.

The larvae and nymphs collected during the survey were reared, yielding 180 adults (111 in Abéché and 69 in Biltine), all identified as *A. aegypti.*

All three risk indices were above WHO thresholds. They were higher in Abéché than in Biltine. However, only the HI showed a significant difference (p=0.004) between the two sites. There was no significant difference for the CI (p=0.6) and BI (p=0.13) index. Stegomyia indices exceed WHO thresholds in Abéché, while in Biltine, the Breteau index remains below the WHO threshold.

### Typology of breeding sites

A variety of larval breeding sites were found, grouped into three categories: flowerpots, water storage containers (jars and barrels), and abandoned objects (tires and containers) (Fig. 2). A total of 126 water containers were counted during the survey. Flowerpots were the most common (69%), followed by water containers (17%) and abandoned objects (14%).

**Figure 2:** A. Flower pots in a household; B. Abandoned container found full of *Aedes aegypti* larvae; C. Water storage containers

### Collection of adult mosquitoes

A total of 40 rooms were sprayed in Abéché and 60 in Biltine. A total of 1,859 female mosquitoes of three genera *(Aedes, Anopheles* and *Culex)* were collected by PSC. The results are presented in Table II.

**Table II:** Distribution of mosquitoes collected by PSC on the two sites

Cities	No. of rooms sprayed	Moustiques femelles collectés/Female mosquitoes collected			
		*Aedes* spp n (%)	*Anopheles* spp n (%)	*Culex* spp n (%)	Total n (%)
Abéché	40	190 (25)	42 (6)	526 (69)	758 (100)
Biltine	60	100 (9)	689 (63)	312 (28)	1 101 (100)
**Total**	**100**	**290 (16)**	**731 (39)**	**838 (45)**	**1 859 (100)**

n = Nombre de moustiques par genre collectés par ville/Number of mosquitoes by genus collected by city

All the 290 *Aedes* specimens collected by PSC were morphologically identified as *A. aegypti.* Thus, for the total collection (PSC and larval collection), there were 470 *A. aegypti* (23% of female mosquitoes).

## Discussion

The objective of the survey was to identify the *Aedes* species likely to transmit VCHIK in the towns of Abéché and Biltine, where the epidemic was declared. The activity reports of the vector control section of the PNLP in 2017 and 2018 had reported the presence of mosquitoes of the genus *Aedes* in Abéché and Biltine, but did not provide details on the species [18, 19]. Our survey confirms the presence of *A. aegypti* in remarkable numbers (23% of mosquitoes collected) in both localities where VCHIK had already been isolated from patients without recent travel history. *Aedes aegypti* is one of the main vectors of VCHIK, along with *A. albopictus* [12]. Its presence and the massive infection of the population suggest that VCHIK is transmitted autochthonously in these cities in eastern Chad. However, its role as a vector in the two cities could not be established due to the lack of adequate sample preservation facilities. Flowerpots accounted for more than two thirds of *Aedes* breeding sites, followed by domestic water storage containers. Because flowers are maintained throughout the year and difficult access to water forces residents to store water, *A. aegypti* is likely to find breeding sites even in the middle of the dry season, as Diarrassouba and Dossou-Yovo [10] found in West Africa. In Abéché, as in Biltine, flower cultivation seems to be the rule among residents, who are probably unaware that flowerpots are potential breeding sites for *A. aegypti.* Households without flowerpots are the exception. Communication to change people's behavior would be very important to slow down the spread of vectors in the region. Insecticide sensitivity testing to optimize chemical vector control could not be conducted. The samples of *A. aegypti* larvae collected were insufficient (only 180 female *A. aegypti* from the larval collection at the two sites) to be considered. In both areas investigated, the three risk indices measured were well above the thresholds defined by the WHO [16]. However, as there is no arbovirus vector surveillance in Chad to sound the alarm, this epidemic came as a surprise to all health system stakeholders, including the epidemiological surveillance service. Risk indices were higher in Abéché than in Biltine. By September, the rain had stopped in Biltine. In Tanzania, Kahamba *et al.* [14] have shown that the HI and BI decrease in the dry season. It is possible that the drying up of certain breeding sites and climatic factors such as high temperatures and low relative humidity had a negative effect on mosquito development in Biltine. Abéché, on the other hand, was surveyed at the height of the rainy season, with lower temperatures and higher relative humidity.

A low proportion of adult *A. aegypti* was collected at both sites (16%). Two thirds of collected *A. aegypti* females were found in Abéché, where the surveyed rooms were 40% of the PSC rooms. The species generally has a strong endophilic tendency, especially in the Neotropics [22, 24]. However, it can be more exophilic in certain areas, as shown by Badolo *et al.* [3] in Burkina Faso. If this was the case at our two sites, the collection of residual endophilic fauna did not enable a proper assessment of vector density. Other sampling methods, such as the use of a CDC backpack vacuum cleaner [7], capture on human bait, or the use of animal bait or chemical attractant traps, could have been used. These would have made an important contribution to understanding the resting behaviour of *A. aegypti* in eastern Chad. However, in the context of a confirmed epidemic, human trapping would have been too risky and unethical. Furthermore, the traps were not part of the collection equipment in our possession during the survey.

At our recommendation, the municipality of Abéché did insect removal in the vehicles on the move for other cities. An awareness campaign was conducted in the affected localities on the elimination of potential breeding sites and the observance of personal preventive measures.

## Conclusion and outlook

In the course of this investigation, we identified *A. aegypti* as a likely vector of CHIKV transmission in the localities we visited. For better vector control, knowledge of vector behavior and susceptibility to insecticides is essential. Due to the insufficient number of samples (only 180 *A. aegypti* females from the larval collection at the two sites), susceptibility tests could not be performed. On the other hand, the rate of CHIKV infectivity in the vectors could not be determined due to lack of suitable storage material. Other questions remain, in particular regarding the genotypic lineage and source of the virus at the origin of this epidemic.

## Funding

This work was carried out with the financial support of the Chad Ministry of Public Health.

## Acknowledgments

We thank the Ministry of Health, through the Directorate of Epidemiological Surveillance, for funding the field activities. We thank the provincial health authorities for their administrative support and the community relays for facilitating household access.

## Authors’ contributions

MOUNDAI Tchonfienet: Study design, literature survey, definition and drafting of methodology, field surveys, data analysis and interpretation of results, and drafting of manuscript.

HAMIT Mahamat Alio: Methodology, data analysis and co-editing of manuscript

DEMBA KODINDO Israël: Field surveys and co-writing of the manuscript

YANGALBE Kalnoné Élise: Methodology and field surveys

KERAH Hinzoumbé Clément: Protocol validation, data analysis and manuscript proofreading and validation

ABDELSALAM Tidjani: Proofreading and validation of manuscript

KEKEUNOU Sévilor: Proofreading and validation of the manuscript

## Links of interest

The authors declare that they have no conflict of interest with the content of this article.
